# The Relationship of Acne With Somatosensory Amplification, Health Anxiety, and Depression Levels

**DOI:** 10.7759/cureus.32314

**Published:** 2022-12-08

**Authors:** Işın Öncü, Gülhan Gürel, Ayşe Akkoyun

**Affiliations:** 1 Department of Dermatology, Kadirli State Hospital, Osmaniye, TUR; 2 Department of Dermatology, Afyonkarahisar Health Sciences University, Afyonkarahisar, TUR; 3 Department of Psychiatry, Kırıkkale University, Kırıkkale, TUR

**Keywords:** somatization, psychiatry, depression, anxiety, acne

## Abstract

Objective

The objective of this study is to evaluate the somatosensory amplification, as well as anxiety and depression levels in acne vulgaris (AV) patients, and to examine their relationship with disease severity.

Methods

Sociodemographic form, Global Acne Grading System (GAGS), Somatosensory Amplification Scale (SSAS), Health Anxiety Inventory (HAI), Beck Anxiety Inventory (BAI), and Beck Depression Inventory (BDI) were applied to the patient group. All scales, except GAGS, were also applied to the healthy controls.

Results

All psychiatric scale scores of acne patients were higher than those of the control group. Moreover, the patient group had significantly higher SSAS, BDI scores, HAI total scores, and subscales of hypersensitivity to somatic symptoms and anxiety compared to the healthy controls. A positive but weak correlation was found between all scale scores. In patients with AV, no correlation was found between acne severity, age, disease duration, and all scale scores.

Conclusion

A significant relationship was found between somatosensory amplification, depression, and health anxiety in acne patients, independent of global acne severity, age, and disease duration. More successful acne treatment and patient management will be possible with an interdisciplinary approach that includes both psychiatry and dermatology.

## Introduction

Acne vulgaris (AV) is a common dermatological disease with limited, chronic, inflammatory, and genetic-hormonal interaction of pilosebaceous units [1]. It is the eighth most common disease in the world and is the chronic skin disease with the longest disease duration after eczema [2,3]. AV affects the areas where hormonally sensitive sebaceous follicles are concentrated on the skin, such as the face [1,4]. Acne lesions are associated with many psychological comorbidities when they are predominately located on exposed areas of the body, such as the face, which has an important place in physical appearance. A decrease in the quality of life, disturbed body image, a decrease in self-esteem, social isolation, stigma, sexual dysfunction, social phobia, anxiety, depression, anger, and suicide attempts have been reported in patients with moderate and severe acne [1,4-7].

Female patients with chronic acne suffer from distress more than men. Moreover, those with facial localization are impacted more than those with trunk localization, and the severity of the disease and the severity of distress follow a parallel course. Emotional stress can occur as a result of acne as well as trigger or exacerbate acne [3,5]. Acne can cause psychological disorders with its clinical manifestations and can be triggered by psychological factors ranging from depression, anxiety, and somatization to psychosis [4]. In AV patients, health anxiety, somatosensory amplification, depression, and anxiety levels may be higher in relation to the severity of the disease.

Since acne can cause cutaneous and psychological scars in the long term, more successful results can be achieved by getting psychiatric support during the application of acne treatment. As far as we know, there are no studies in the literature that evaluated the somatosensory amplification and the level of health anxiety in patients with acne.

The aim of this study was to evaluate factors such as somatosensory amplification, anxiety, and depression levels in AV patients and to examine their relationship with disease severity.

## Materials and methods

This is a case-control, observational study evaluating patients admitted to the dermatology outpatient clinic due to acne between May 2020 and March 2021. One hundred twenty-three patients between the ages of 18 to 45 years participate in the study. The control group constituted of 100 healthy volunteers. In May 2020, the proposal for this study was approved by the Institutional Review Board of Afyonkarahisar Health Sciences University.

The inclusion criteria of the study were patients aged between 18 to 45 years with a diagnosis of AV, who volunteered to participate in the study by signing the informed consent and had the ability to read and write. Exclusion criteria were ages <18 or >45, having a history of comorbid diseases, having neurological or psychiatric diseases, and receiving systemic treatment for AV.

The study was conducted in accordance with the principles of the Declaration of Helsinki. Written informed consent form was obtained from each participant.

All patients were asked to fill out a sociodemographic form, which included information about age, gender, educational status and occupation, Somatosensory Amplification Scale (SSAS), Health Anxiety Inventory (HAI), which assesses the severity of health anxiety, Beck Anxiety Inventory (BAI), which measures anxiety level, and Beck Depression Inventory (BDI), which measures depression symptoms such as unhappiness, pessimism, and reluctance. These scale values were compared with healthy controls. In patients diagnosed with acne vulgaris, acne severity was evaluated by a dermatologist according to the Global Acne Grading System (GAGS).

Somatosensory Amplification Scale (SSAS)

This scale was developed by Barsky et al. to measure how individuals experience somatic symptoms and their susceptibility to somatization [8]. It is a five-point Likert scale. Bodily sensations that do not indicate a certain disease are questioned with 10 items. The total score ranges between 10 and 50. The validity and reliability of the Turkish version was evaluated by Güleç et al. [9].

Health Anxiety Inventory (HAI)

HAI was developed by Salkovskis et al. to evaluate health anxiety from a cognitive and emotional perspective [10]. It is a four-point Likert scale. While 14 out of 18 questions inquire about the current situation, when answering the last four questions the respondents are asked to assume that they have a more serious disease. Karaer et al. and Aydemir et al. performed two separate validity and reliability studies in the Turkish population [11].

Beck Anxiety Inventory (BAI)

BAI, which was created by Beck et al., is a four-point Likert-type, 21-item scale used to measure the severity of anxiety in children and adults [12]. The total score of 0-7 is interpreted as minimal anxiety, 8-15 as mild anxiety, 16-25 as moderate anxiety, and 26-63 as severe anxiety. Turkish validity and reliability study was conducted by Ulusoy et al. in 1998 [13].

Beck Depression Inventory (BDI)

Developed by Beck et al. in 1961, BDI aims to measure somatic, affective, cognitive, and motivational symptoms along with depression level and severity. It is a four-point Likert-type scale consisting of 21 questions [14]. The total score of 0-13 indicated minimal depression, 14-19 mild, 20-28 moderate, and 29-63 severe depression. Turkish validity and reliability study was done by Hisli et al. The cut-off score is accepted as 17 [15].

## Results

Among the patients with acne, 85 (69.1%) were female, and 38 (30.9%) were male, with a mean age of 24.7±3.1 years. In the control group, 60 (60.0%) individuals were female, and 40 (40.0%) were male, with a mean age of 25.3±4.0 years. The mean age and sex ratios of the patient and control groups were similarly distributed (p=0.219 and p=0.202 for each). Demographic and clinical characteristics of patients and controls are shown in Table 1. Acne was mild in 44.7% of the patients, moderate in 32.5%, and severe in 22.8%. The median duration of illness was 12.0 [0.2- 240.0] months.

**Table 1:** Comparison of some demographic and clinical parameters of the patient and control groups * The t test was used for independent groups. Descriptive statistics were given as mean ± standard deviation ** Pearson Chi-Square/Fisher Freeman Halton test was used. Descriptive statistics were given as number, (%) *** Descriptive statistics are given as median [minimum-maximum] P-values of p<0.05 were considered statistically significant

	Control (n=100)	Patient (n=123)	p-value
Age	25.3 ± 4.0	24.7 ± 3.1	0.219*
Gender (%)			
Female	60 (60.0)	85 (69.1)	0.202**
Male	40 (40.0)	38 (30.9)	
Marital status (%)			
Married	33 (33.0)	20 (16.3)	0.004**
Single	65 (65.0)	102 (82.9)	
Widowed/ divorced	2 (2.0)	1 (0.8)	
Education status (%)			
Elementary school	1 (1.0)	0 (0.0)	0.187**
Middle school	4 (4.0)	5 (4.1)	
High school	16 (16.0)	32 (26.0)	
Bachelors/ Masters/ PhD	79 (79.0)	86 (69.9)	
Occupation (%)			
Student	25 (25.0)	61 (49.6)	0.001**
Civil servant	59 (59.0)	40 (32.5)	
Worker	2 (2.0)	6 (4.9)	
Self-employed	4 (4.0)	5 (4.1)	
Housewife	5 (5.0)	5 (4.1)	
Unemployed	5 (5.0)	6 (4.9)	
Global Acne Severity	-	21.0 ± 8.4	NA
Global Acne Severity (%)			
Mild	-	55 (44.7)	NA
Moderate	-	40 (32.5)	
Severe	-	28 (22.8)	
Disease duration (months)	-	12.0 [0.2- 240.0]	NA***
Benefit from previous treatments (%)			
Yes	-	35 (28.5)	-
No	-	88 (71.5)	

Table 2 shows the comparison of the mean scores for total SSAS, HAI total score and subscales scores, BAI, and BDI scores between the groups. All psychiatric scale scores of the patients presenting with acne were higher than those of the control group. The patient group had significantly higher mean scores of SSAS, total score of HAI and subscales of hypersensitivity to somatic symptoms and anxiety, and BDI scores than controls (p=0.002, p<0.001, p=0.006, respectively). When the results of BAI and HAI subscales of negative consequences were evaluated according to the groups, the differences between the scores were not significant (p=0.070, p=0.862, respectively).

**Table 2:** Comparison of the scales used in the patient and control groups * The t-test was used for independent groups. Descriptive statistics were given as mean ± standard deviation ** The Mann-Whitney U test was used. Descriptive statistics are given as median [minimum-maximum] *** Pearson Chi-Square/Fisher Freeman Halton test was used. Descriptive statistics were given as number, (%) P-values of p<0.05 were considered statistically significant

	Group		
	Control (n=100)	Patient (n=123)	p-value
Somatosensory Amplification Scale - total score	26.1 ± 8.6	29.5 ± 7.3	0.002*
Health Anxiety Inventory - total score	13.9 ± 6.5	17.3 ± 7.1	<0.001*
Hypersensitivity to somatic symptoms and anxiety	10.4 ± 5.7	13.7 ± 5.8	<0.001*
Negative consequences	3.0 [0.0- 12.0]	3.0 [0.0- 10.0]	0.862**
Beck Anxiety Inventory - total score	9.0 [0.0- 47.0]	11.0 [0.0- 52.0]	0.070**
Beck Anxiety Inventory (Catg.) (%)			
Minimal	45 (45.0)	45 (36.6)	0.423***
Mild	32 (32.0)	38 (30.9)	
Moderate	12 (12.0)	22 (17.9)	
Severe	11 (11.0)	18 (14.6)	
Beck Depression Inventory - total score	6.0 [0.0- 36.0]	8.0 [0.0- 53.0]	0.006**
Beck Depression Inventory (Catg.) (%)			
Minimal	72 (72.0)	72 (58.5)	0.187***
Mild	16 (16.0)	33 (26.8)	
Moderate	8 (8.0)	11 (8.9)	
Severe	4 (4.0)	7 (5.7)	

In the analysis of the relationship between the scale scores and patients' global acne severity, disease duration and age (Table 3), there was no significant linear correlation between SSAS, HAI total and subscales, BAI and BDI scores, and global acne severity, age, and duration of complaints (p>0.05 for all). The duration of complaints increased with increasing age (r=0.315, p<0.001).

**Table 3:** Global acne severity; correlation between disease duration, age, and scales * Pearson correlation coefficient was used ** Spearman's Rho Correlation coefficient was used P-values of p<0.05 were considered statistically significant

Patient group	Global Acne Severity		Age		Disease duration	
	r	p-value	r	p-value	r	p-value
Age	-0.167	0.065*	-	-	-	-
Disease duration	-0.016	0.861*	0.315	<0.001*	-	-
Somatosensory Amplification Scale	-0.059	0.514*	0.135	0.138*	0.124	0.170**
Health Anxiety Inventory - total score	-0.092	0.309*	-0.121	0.182*	0.021	0.821**
Hypersensitivity to somatic symptoms and anxiety	-0.081	0.371*	-0.127	0.162*	-0.005	0.956**
Negative consequences	-0.074	0.414**	-0.027	0.769**	0.1	0.271**
Beck Anxiety Inventory - total score	-0.014	0.878**	0.028	0.762**	0.04	0.662**
Beck Depression Inventory - total score	0.058	0.524**	0.037	0.688**	-0.01	0.913**

In the last stage of the study, all scales were compared within the patient group. There was a significant, linear, same-sided, weak relationship between the patients' SSAS scores and HAI total, BAI, and BDI scores (p<0.001 for all). A positive and strong correlation was found between the total HAS scores of the individuals in the patient group and hypersensitivity to somatic symptoms, anxiety, and negative consequences subscales (r=0.954, p<0.001 and r=0.672, p<0.001, respectively). A positive, moderate correlation was found between BAI total score and BDI scores in the patient group (r=0.587, p<0.001). The results of the analyses are presented in Table 4.

**Table 4:** Correlation between scales * Pearson Correlation coefficient was used ** Spearman's Rho Correlation coefficient was used P-values of p<0.05 were considered statistically significant

Patient group		r	p-value
Somatosensory Amplification Scale	Health Anxiety Inventory - total score	0.439	<0.001*
Somatosensory Amplification Scale	Hypersensitivity to somatic symptoms and anxiety	0.424	<0.001**
Somatosensory Amplification Scale	Negative consequences	0.185	0.040**
Somatosensory Amplification Scale	Beck Anxiety Inventory - total score	0.477	<0.001**
Somatosensory Amplification Scale	Beck Depression Inventory - total score	0.362	<0.001**
Health Anxiety Inventory - total score	Beck Anxiety Inventory - total score	0.355	<0.001**
Health Anxiety Inventory - total score	Beck Depression Inventory - total score	0.392	<0.001**
Hypersensitivity to somatic symptoms and anxiety	Negative consequences	0.369	<0.001**
Hypersensitivity to somatic symptoms and anxiety	Beck Anxiety Inventory - total score	0.346	<0.001**
Hypersensitivity to somatic symptoms and anxiety	Beck Depression Inventory - total score	0.369	<0.001**
Negative consequences	Beck Anxiety Inventory - total score	0.223	0.013**
Negative consequences	Beck Depression Inventory - total score	0.271	0.002**
Beck Anxiety Inventory - total score	Beck Depression Inventory - total score	0.587	<0.001**

## Discussion

The results of our study show that individuals with AV exhibit more somatization, depression, hypersensitivity to somatic symptoms, and anxiety symptoms compared to the control group. Somatosensory amplification in patients with AV may be associated with hypersensitivity to somatic symptoms, anxiety, health anxiety, and depression. Acne can lead to psychological disorders, and even if the acne is not severe, it can be perceived as more severe, which may cause more anxiety and depression.

The prevalence of psychiatric comorbidities in AV patients varies between 35.5% and 38.5% [16]. In a study conducted with the Skindex-16 quality of life questionnaire, the scores of acne patients were higher than the scores of patients with inflammatory skin diseases such as eczema and psoriasis. Emotional scores were high even in patients with only a few comedones [17]. Acne is one of the most common dermatological diseases and can affect people not only physically but also psychosocially. Along with providing appropriate dermatological medical treatment, it is very important to detect psychiatric problems and to increase awareness of such issues. Successful medical treatment for acne can improve the individual's appearance and lesions, while also improving comorbidities such as depression and anxiety.

According to the Global Burden of Disease study, AV affects ~85% of young adults aged 12-25 years [2]. Although many acne-related psychosocial disorders have been identified in adolescence, studies investigating adult acne-related psychosocial disorders are insufficient [18]. Aksu et al. conducted a study that included 2300 students with AV with a mean age of 15.10±1.53 years. In the 13-14 years age group, girls with AV were statistically more affected than boys, and in the 15-18 age group, boys with AV were statistically more affected than girls. Acne severity has been associated with disease duration and age [19]. In their study involving 171 patients with AV, Hayta et al. did not observe a significant difference in acne severity in different age groups [20]. Gul et al. conducted a study to investigate the psychiatric conditions and personality traits of 40 adult acne patients and found that the mean age of the patients was 28.87±4.73 years [21]. In a large-scale study aimed at revealing the psychosocial impact of acne involving 213 acne patients and 213 controls, Altunay et al. found that the mean age was 24.4±7.1 years [18]. In our study, the mean age of the patient group consisting of late adolescents and young adults was 21.4±3.81 years, which was similar to the literature. Although acne is typically considered to be a disease of the adolescent age group, the findings obtained from research and clinical applications in the last two decades have shown that acne is common in the adult population, which supports our findings. There was no significant relationship between the severity of acne and age. It was found that the duration of the disease increased with age.

Most studies have found that severe acne is more common in men than in women. Hayta et al. reported that male patients had more severe acne than female patients [20]. Similarly, Aksu et al. found more severe acne in men [19]. However, in other large-scale studies with AV patients, there was no significant difference between genders in terms of acne severity [22,23]. In our study, we also did not detect any significant relationship between acne severity and gender.

Aksu et al. found that the duration of the disease and the severity of acne increased in patients with AV [19]. Yarpuz et al. evaluated the correlation between acne severity and disease duration in patients with AV and did not find any correlation [22]. In our study, there was no significant relationship between the severity of acne and disease duration.

To the best of our knowledge, there is no study in the literature evaluating SSAS and HAI scores in patients with acne. In our study, the SSAS and HAI scores were significantly higher in acne patients compared to the control group. However, no significant correlation was found between age, acne severity, disease duration, and SSAS and HAI scores.

Although health anxiety is not a somatic disease, it is defined as a situation in which, depending on their perceptions, individuals believe that they have a serious illness. SSAS eliminates the subjective judgment of physicians, allowing for the measurement of the intensity of the various somatic symptoms that patients complain about. Increases in health anxiety have been correlated with increases in depression and anxiety [24]. Some studies have reported a positive association between depression, anxiety, and somatosensory amplification. This relationship can be especially seen when a person perceives somatic sensations as more intense, harmful, and worrying [25]. In addition, somatic symptoms are a behavioral model that can be used by individuals to gain support and strength from their social environment and family [26]. From this point of view, it can be thought that patients with acne engage in such behaviors to gain strength and support from their families and communities and to reduce their sense of inadequacy.

Gul et al. evaluated 40 adult acne patients with somatization, depression, and anxiety subscales of the psychological symptom screening list (SCL-90-R) and reported higher somatization, anxiety, and depression scores in the patient group. They suggested that high somatization scores resulted in a higher rate of psychosomatic illness experience in patients with acne [21]. In a meta-analysis study on hypochondriasis and somatization, which included 47 studies, Creed et al. concluded that there is a significant relationship between somatosensory amplification and somatization [27]. However, in a study with 150 adults with acne and 50 healthy controls, Hafez et al. used SCL-90-R, and no significant difference between the groups was found in somatization scores [28]. In a multicenter study using the body dysmorphic disorder questionnaire in 245 patients with AV, it was found that the prevalence of body dysmorphic disorder was high in patients with acne. Ninety-five percent of patients reported that the part of their body that worries them the most was their face [29].

Based on the literature, some of the acne patients were diagnosed with somatization disorder, while some of them had higher somatization scores than the control group. There were no studies evaluating the relationship between somatosensory amplification and depression and anxiety in patients with AV in the Pubmed database. Apart from dermatological diseases, SSAS as well as anxiety and/or depression scales have been investigated in chronic diseases of unknown etiology, such as migraine, vertigo, and male infertility. Öztekin et al. evaluated 130 infertile male patients and found that the mean scores of SSAS, HAI, BAI, and BDI were significantly higher in the infertility group than in the control group. They commented that patients with high levels of health anxiety and increased bodily sensations may visit hospitals more frequently, which leads to unnecessary detailed examinations causing both economic and labor loss, which in turn may further increase the already high levels of anxiety and depression in these patients [30].

Studies evaluating the relationship between acne and anxiety and depression reveal conflicting results [3,4,6,16,31,32]. Depression has been reported in 5.6%-8.8% of AV patients. Compared to the general population, the rate of depression was two to three times higher in AV patients. In some studies, the presence of anxiety in AV patients was reported at highly variable rates, such as 4.35%-68.3%, and higher than in control groups [3]. Some studies have reported that anxiety and depression are not associated with age and gender, while other psychiatric studies have reported that these psychiatric disorders are more common in women [32]. Although anxiety appears to be a more common problem than depression in most studies, the prevalence of anxiety and depression varies widely. These inconsistencies may be mostly due to the differences in the adolescent population, variation in the interpretation of the scale scores, differences in the predictive cut-off values, use of a different screening tools for depression/anxiety, and/or the different sociocultural characteristics of the countries [18]. In a study conducted by Yazici et al. using the Hospital Anxiety and Depression Scale (HADS) in 61 patients with AV, the frequency of depression was 29.5%, while that of anxiety was 26.2%, and these differences were significant compared to healthy controls. They reported that there was no significant relationship between the anxiety and depression scores of AV patients and their gender and disease severity [33]. Aktan et al. evaluated 615 acne patients using the HADS. While the frequency of anxiety and depression in the acne group was 24.7% and 13.3%, respectively, they were 25.2% and 15.2% in the control group. The HADS anxiety subscale scores of the females in the acne group were significantly higher than those of the males. They did not find a significant relationship between anxiety and depression scores and disease severity in patients with AV. The authors commented that in adolescence, the effects of cosmetic appearance, stigma, and acne on body image are more important for girls since they are more vulnerable psychologically than boys [34].

In a study conducted with 82 patients with acne using HADS, Golchai et al. reported higher anxiety scores in the acne group compared to the control group, although the depression scores were not significantly different. They did not find a significant relationship between the presence and severity of anxiety and factors such as age, gender, marital status, or scar formation. The authors interpreted that the presence of even mild lesions in important body areas such as the face may be disturbing and may appear larger for the patient and may eventually cause anxiety due to individual body image perceptions [32]. Hayta et al. applied BDI to 171 patients with AV and investigated the relationships between age, gender, and acne severity. Although there was no significant relationship between the presence of depression and acne severity and age, they found that women were more depressed than men [20].

Altunay et al. used HADS to investigate the psychosocial effects of acne in 213 adult acne patients and found that anxiety and depression were not associated with acne severity or disease duration but were associated with the level of disease-related anxiety [18]. In a recent meta-analysis of 42 studies, depression and anxiety were reported to be more common in individuals with acne than in controls in adolescence but more frequently in adulthood. Moreover, depression has been reported to be approximately twice as common in women with AV as in men [7].

Although many studies on acne and depression have stated that the prevalence of depression increased in acne patients, a few stated that there was no relationship. Some studies have reported that anxiety and depression were not associated with age and gender. Some studies have reported that anxiety is more common in women, those with an early onset of the disease, those with a long-term disease, and those with more severe acne. On the other hand, some other studies reported no relationship between acne and anxiety and between acne severity and the severity of anxiety and depression [3,32].

In our study, no significant correlation was found between the presence and severity of anxiety and depression and factors such as age, acne severity, and disease duration. However, there was a correlation between BAI and BDI scores and all other scale scores, including SSAS scores. Based on these findings, we believe that patients with AV may perceive mild or localized lesions as more severe. This exaggerated somatization in patients with acne can be caused by psychiatric disorders such as depression and anxiety.

Our study contributes to the literature with valuable data suggesting that somatosensory amplification is significantly associated with health anxiety and symptoms of depression in patients with AV, however, there are some limitations. The cross-sectional design is one of these limitations. Another limitation of the study is that the forms used to evaluate anxiety, depression, and somatosensory amplification were based on patients' own reports. Although self-report forms are functional in time-limited situations, they may fail to assess the general condition of the individual as they usually reflect the individual's momentary state. In addition, the number of female participants was higher than male participants. However, these figures reflect the real-life situation because the rate of admission to the clinic due to AV in female patients is higher than in male patients. Large-scale studies are needed to generalize the results of this study.

## Conclusions

Since at least one-third of the patients seen in dermatology clinics present with significant psychological complaints and acne is frequently encountered in dermatology outpatient clinics, it is very important for dermatologists to be aware of such possibilities and to detect psychiatric problems. Because acne is a chronic disease, patients may be exposed to prolonged treatment and repeated medical examinations. This process can lead to depression, somatization, and increased health concerns. In patients who admitted with somatic complaints, these complaints may be related to reasons such as somatosensory amplification, depression, and health anxiety. More successful acne treatment and patient management will be possible with an interdisciplinary approach that includes psychiatry and dermatology. Long-term, prospective, randomized studies are needed for further studies.
